# The Etiology of Neuromuscular Hip Dysplasia and Implications for Management: A Narrative Review

**DOI:** 10.3390/children11070844

**Published:** 2024-07-11

**Authors:** Ana Presedo, Erich Rutz, Jason J. Howard, Michael Wade Shrader, Freeman Miller

**Affiliations:** 1 Department of Pediatric Orthopaedics, Robert Debré University Hospital, 75019 Paris, France; ana.presedo@aphp.fr; 2 Department of Orthopaedics, The Royal Children’s Hospital, Melbourne 3052, Australia; erich.rutz@rch.org.au; 3 Murdoch Children’s Research Institute, Melbourne 3052, Australia; 4 Department of Paediatrics, The University of Melbourne, Melbourne 3010, Australia; 5 Medical Faculty, University of Basel, 4001 Basel, Switzerland; 6 Department of Orthopaedics, Nemours Children’s Health, Wilmington, DE 19803, USA; jason.howard@nemours.org (J.J.H.); wade.shrader@nemours.org (M.W.S.)

**Keywords:** hip development, neuromuscular hip dysplasia, cerebral palsy, spastic hip, hypotonic hip

## Abstract

This study summarizes the current knowledge of the etiology of hip dysplasia in children with neuromuscular disease and the implications for management. This article is based on a review of development of the hip joint from embryology through childhood growth. This knowledge is then applied to selective case reviews to show how the understanding of these developmental principles can be used to plan specific treatments. The development of the hip joint is controlled by genetic shape determination, but the final adult shape is heavily dependent on the mechanical environment experienced by the hip joint during growth and development. Children with neuromuscular conditions show a high incidence of coxa valga, hip dysplasia, and subluxation. The etiology of hip pathology is influenced by factors including functional status, muscular tone, motor control, child’s age, and muscle strength. These factors in combination influence the development of high neck–shaft angle and acetabular dysplasia in many children. The hip joint reaction force (HJRF) direction and magnitude determine the location of the femoral head in the acetabulum, the acetabular development, and the shape of the femoral neck. The full range of motion is required to develop a round femoral head. Persistent abnormal direction and/or magnitude of HJRF related to the muscular tone can lead to a deformed femoral head and a dysplastic acetabulum. Predominating thigh position is the primary cause defining the direction of the HJRF, leading to subluxation in nonambulatory children. The magnitude and direction of the HJRF determine the acetabular shape. The age of the child when these pathomechanics occur acts as a factor increasing the risk of hip subluxation. Understanding the risk factors leading to hip pathology can help to define principles for the management of neurologic hip impairment. The type of neurologic impairment as defined by functional severity assessed by Gross Motor Function Classification System and muscle tone can help to predict the risk of hip joint deformity. A good understanding of the biomechanical mechanisms can be valuable for treatment planning.

## 1. Introduction

A complex combination of genetic programming and response to the physical environment is required for normal human development. One of the best studied and most widely recognized developmental processes is the formation of normal eyesight. Genetics guides the formation of the gross physical eye and brain development and connections; however, at a critical stage of infancy and early childhood, functional sight will not develop unless light and physical vision are available to the eye. This has been clearly demonstrated by the need to remove congenital cataracts early; otherwise, normal sight does not develop [1]. This has led to a focus on early screening for congenital cataracts [2]. Like the requirement for normal functional vision, the hip requires normal stress with normal muscle forces and age-appropriate gait to develop normal shape and function. As with treatment of the eyes, the early identification and treatment of childhood hip pathology have become an increasing focus. The diagnosis of developmental hip dysplasia (DDH) can also be made at birth. Screening and early treatment lead to higher normal hip development [3]. There are many factors influencing the etiology and development of DDH; however, swaddling the baby with the hips adducted and extended for prolonged periods as a common cultural practice has been shown to be one important factor [4]. Hip dysplasia (Supplementary Table S1) is very common in children with neurologic impairment. In patients with cerebral palsy (CP), for example, the early identification of hip displacement constitutes a major focus of orthopedic management to achieve an optimal long-term outcome [5,6]. These early screening efforts have clearly identified a population of young children with severe neurologic impairment as being at the highest risk of hip pathology [5,7]. These neurologic impairments cause abnormal hip postures and delayed gross motor function such as standing and walking. This exposes the developing hip to the impact of abnormal forces acting on a cartilage model, which determines the final morphology of the bone structure [5]. The normal shape of the proximal femur and acetabulum is important to the formation of a stable hip [8]. Children with neurologic impairments ranging from high to low muscular tone show a high incidence of increased femoral neck–shaft angle (NSA) and femoral anteversion (FA) [5]. The primary measurement method to determine hip displacement for monitoring and planning intervention of neuromuscular hip dysplasia is the Reimers’ migration index or migration percentage (MP) (Supplementary Table S1) [9]. Other parameters are also taken into account: (a) the angle between the axis of the femoral neck and the axis of the femoral shaft in the frontal plane called the NSA and (b) the angle of rotation of the femoral neck relative to the diaphysis in the transverse plane commonly called FA (Supplementary Table S1) [8,10]. Many childhood hip pathologies and their treatment involve altering the shape of the proximal femur, either the NSA or the FA. The importance of the head–shaft angle (HSA) has also been pointed out in the literature [11,12]. It is measured as the angle between a line perpendicular to the proximal femoral physis and a line through the middle of the femoral shaft on an anteroposterior radiograph of the pelvis. The HSA was first described to assess slipped capital femoral epiphysis by Southwick in 1967 [1] and later applied to the CP hip by Foroohar et al. in 2009 [13].

The goal of this study was to summarize scientific knowledge regarding the etiology of the proximal femoral shape and hip joint development during childhood growth. Treatment planning utilizing this developmental understanding will be discussed using selected cases. 

## 2. Materials and Methods

The initial review included an extensive literature search related to the embryology of hip development. These data were used as the framework for describing normal hip development. A search for the mathematical modeling of hip forces found papers related to understanding how these forces guide further hip development during childhood growth. The search did not recover any information on modeling forces in the infant and how this might influence development. 

## 3. Results

### 3.1. Normal Hip Development

#### 3.1.1. Proximal Femoral Development

**Figure 1:**
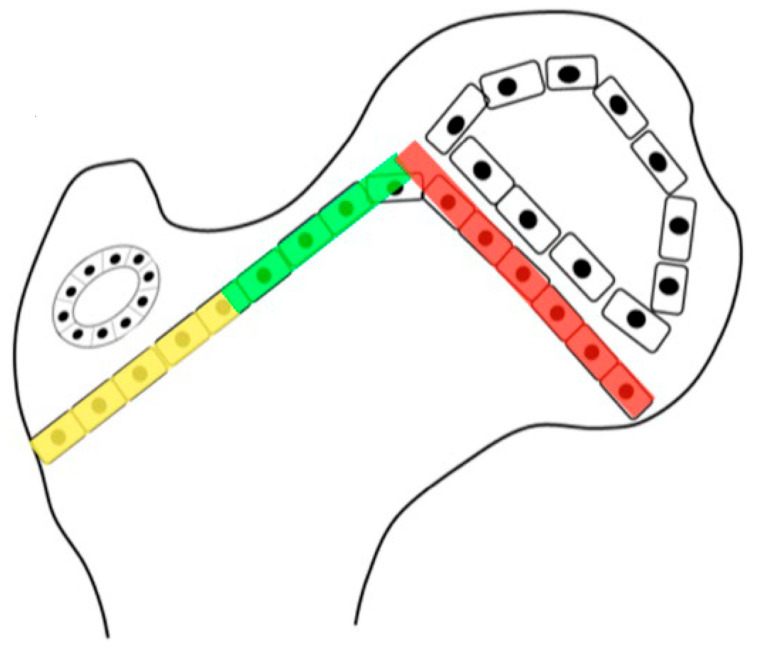
The growth plate of the proximal femur is divided into three sections. The lateral aspect (yellow) is an apophyseal growth plate stimulated by tension and grows laterally and longitudinally. The intermediate growth plate has longitudinal femoral growth (green), and capital epiphyseal growth plate (red) aligns to the hip joint reaction force responding to compression loading. This whole proximal growth plate remains active for longitudinal growth until skeletal maturity, although it contributes to a lower percentage of longitudinal growth as the child ages. Initially up to the age of 5 years, 50% of longitudinal growth comes from the proximal femur, but this gradually decreases to 10% at maturity.

The femoral shape can be recognized first during Carnegie stages (CSs) 17 and 18, which is week 6 of embryonic development. At CS 22–23 (week 8), the femoral head and greater and lesser trochanter can be identified with magnetic resonance imaging. During fetal development, the femoral diaphysis ossification starts mid-femur at a crown-rump length of 40 mm (week 10–11) when the femur is 4–5 mm long [14]. The proximal femoral growth plate is a single continuous plate with the medial side being an epiphysis, and the lateral side is an apophysis, which ossifies later around 48 months of age. The intermediate growth plate connecting these two continues to contribute to longitudinal growth until the end of skeletal maturity but does not develop an ossific cover (Figure 1). Suzuky et al. reported that the NSA started at 140° prior to ossification and then reduced to 130° with a standard deviation of less than 5° until the end of the second trimester of development [15]. Because of the low in utero forces experienced by the developing femur and the low variability, this parameter appears to be primarily driven by genetic shape encoding [16]. Using the Procrustes shape analysis technique, there was no change in the relationship between the location of femoral head and greater and lesser trochanter for the whole developmental period, which is consistent with the stability of the NSA [15]. The NSA also decreases during normal childhood growth from infantile 140–130° to 130–120° at adulthood [17]. The data to support the change in childhood NSA are more limited, with numbers reported by Bobroff et al. [17] actually being cited from a paper by Zipple from 1971 [18]. In a comprehensive review of the current literature, Scorcelletti et al. found significant variation in measurement landmarks in the reported literature for both the NSA and anteversion pointing to the importance of clearly defining these anatomic measurement landmarks. This measurement variability likely explains the variation in reported measurements [19]. 

**Figure 2:**
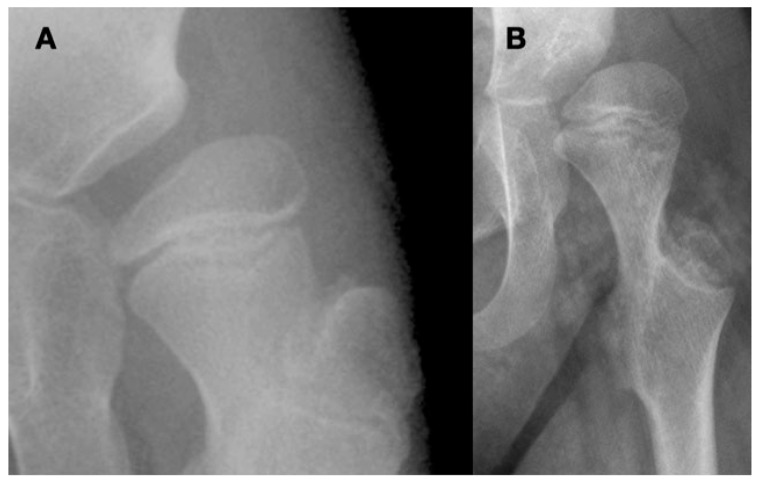
This is a 10-year-old child (**A**) with spastic cerebral palsy, GMFCS V, whose hip demonstrates the common changes in the lateral and posterior openings of the acetabulum, flattening of the medial side of the femoral head, and lateral femoral head overgrowth. The lateral side of the femoral head also shows lower bone density than the medial side. The changes are due to the limited range of motion and a femoral head that is not contained in the opened acetabulum, which is the result of hip joint reaction force focused on the lateral acetabulum and medial femoral head. This 11-year-old child (**B**) is GMFCS III with severe weakness and hypotonia but is able to cruise along furniture and use a walker. His acetabulum is shallow and small with poorly defined edges, and the femoral head is round, has a completely horizontal epiphyseal plate, and is larger than the acetabulum. The femoral neck is in severe valgus and is very thin and long. GMFCS, Gross Motor Function Classification System.

Close interaction of the femoral head and the acetabulum is important for the spherical development of the femoral head and determines its size. Femoral heads that are not completely contained in the acetabulum tend to enlarge and develop less sphericity (Figure 2). 

**Figure 3:**
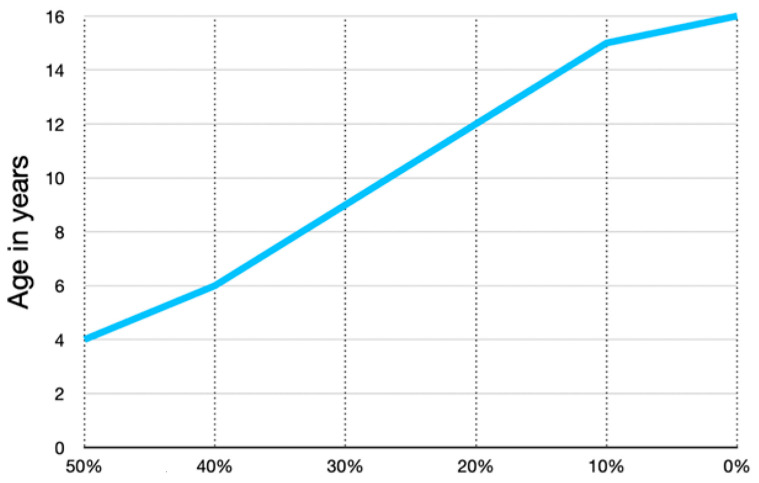
The proportion of longitudinal growth contributed by the proximal femoral growth plate versus the distal is not documented from birth to 4 years of age. We have one case where the growth is documented by pamidronate lines; from age 3–4 years, there was an equal amount of proximal growth compared with distal growth. This graph was independently created using data presented by Pritchett [20] from age 6 to maturity showing that the proximal femur progressively contributes a smaller percentage of total femoral growth with increasing age. This likely accounts for the more rapid proximal femoral shape changes in early childhood.

The percentage of growth in the proximal versus the distal end of the femur between birth and 5 years of age is not well defined; however, it is likely that the proximal femur generates at least 50% of femoral longitudinal growth in these early years (Figure 3). Published data show the 50/50% proximal/distal femoral growth from age 5 years gradually reducing to 10/90% by late adolescence (Figure 3). This rapid early proximal growth can explain why reoccurrence of coxa valga is much faster in the younger ages. 

#### 3.1.2. Development of Anteversion

During fetal development, FA starts at 23° and reduces at the initial femoral shaft ossification stage to slight retroversion (week 10–11), but then again develops anteversion to 25° by 180 mm ossified femoral length. The FA is highly variable with a standard deviation of 20° during the early ossification period [15]. This variability suggests less stable genetic encoding for femoral torsion. During the last trimester, the FA may increase up to 30°, and then, there is a decrease during childhood to a normal FA of between 10 and 20° at adulthood [17]. Since the NSA is less variable than the FA, it is not clear if this difference is mainly related to genetic control or to a higher sensitivity of FA to force exposure. 

#### 3.1.3. Acetabular Development

**Figure 4:**
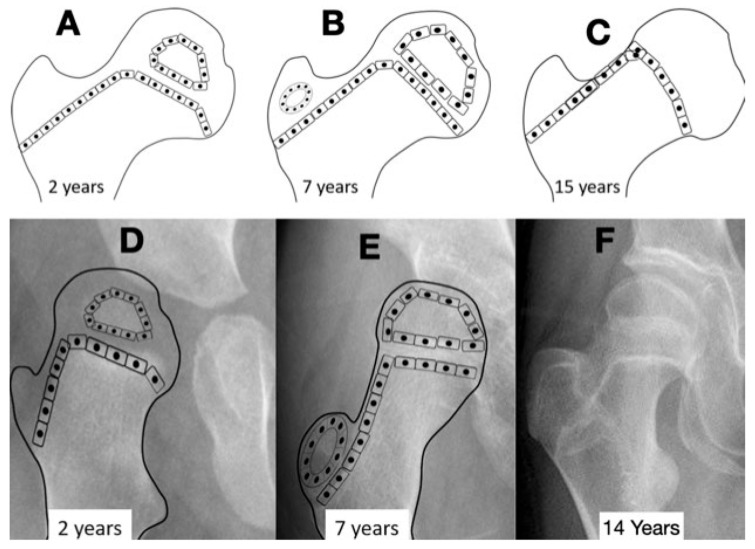
The normal growth plate of the developing femur in a 2-year-old child (**A**), a 7-year-old child (**B**), and an adolescent (**C**) has apophyseal and epiphyseal segments that are in continuity with the intermediate epiphysis. The triangular-shaped epiphyseal plate has differential growth with a relatively slower growth of the apophyseal side compared with the epiphyseal side, creating a more sharply angled growth plate. This difference in apophyseal versus epiphyseal growth in children with cerebral palsy compared with the typically developing child explains the observed difference in proximal femoral shapes (**D**,**E**). The intermediate growth plate contributes to longitudinal femoral growth and width of the femoral neck as shown in this girl, Gross Motor Function Classification System level V, who was treated with pamidronate at age 8 years, leaving a dense line; this radiograph was taken at the completion of growth at age 14 years (**F**). The shape of the growth plate is unchanged until the end of growth. The acetabulum, however, has increased lateral ossific growth as the ring apophysis is ossified. This lateral growth, combined with the femoral head having symmetric medial and lateral growths, has reduced the migration percentage from 28% at age 8 years to 20% at age 14 years (**F**).

Formation of the acetabulum is closely linked to the femoral head formation. When the initial cleft separating the acetabulum and femoral head develops at week 9, the shape is irregular, and by week 11, a round femoral head and acetabulum are present [21]. It has been demonstrated in chicken embryo studies that movement is required for normal spherical hip joint development [18]. The depth and volume of the acetabulum are influenced by the medial pressure applied to the acetabulum by the motion of the femoral head based on studies in lambs and rabbits [22,23]. The radiologic appearance of enlargement of the ossified acetabulum occurs during adolescence by the ossification of the ring apophysis, which decreases MP from age 8 to 12 years by 10 MP (Figure 4).

### 3.2. Biomechanical Considerations

#### 3.2.1. Normal Development

The development of the proximal femur depends on a combination of genetic factors, metabolic growth factors, and mechanical forces [15]. There are also bone pathologies such as osteogenesis imperfecta and osteochondrodysplasias that cause significant proximal femoral alteration due to inherent bone and cartilage pathology. The focus of this paper is the hip in children who are neurologically impaired and do not have primary metabolic bone disease or structural bone or cartilage pathology. Although many children with neurologic impairment, especially those who are nonambulatory, have decreased bone mass density and some may develop bone fragility, there is no published evidence that these changes influence hip joint development. For the remainder of this paper, we will presume the children have bone and cartilage structure and function that responds normally to the applied forces. Therefore, to understand the neurologic hip pathology, we consider the force environment to be the major driver of femoral shape alteration compared with the normal age-matched femoral configuration. This force environment appears to be more important at an earlier age, likely due to the higher percentage of longitudinal growth occurring in the proximal versus the distal femur. 

**Figure 5:**
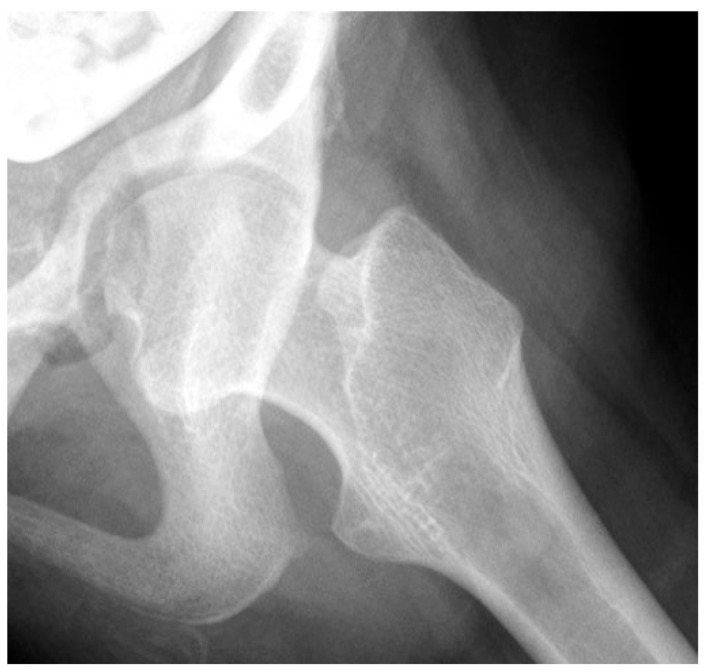
This 16-year-old adolescent with Gross Motor Function Classification System level V cerebral palsy had a severe windblown deformity with fixed right hip abduction, external rotation, and extension contracture, causing the development of acetabular protrusio due to the high medial pressure of the femoral head in the acetabulum.

Close interaction of the femoral head and the acetabulum is important for the spherical development of the femoral head and determines its size. Femoral heads that are not completely contained in the acetabulum tend to enlarge and develop less sphericity (Figure 2). If the acetabulum does not receive pressure from the femoral head, the triradiate cartilage will start to grow laterally and lose the acetabulum sphericity (Figure 3). However, in the case of hyperabducted hips, excessive acetabular depth may develop into a protrusio acetabulum due to the high medially directed force of the femoral head in the acetabulum (Figure 5). Therefore, children with neurologic impairment may have abnormal development of both the proximal femur and acetabulum.

#### 3.2.2. Biomechanical Principles Driving Femoral Shape Development

**Figure 6:**
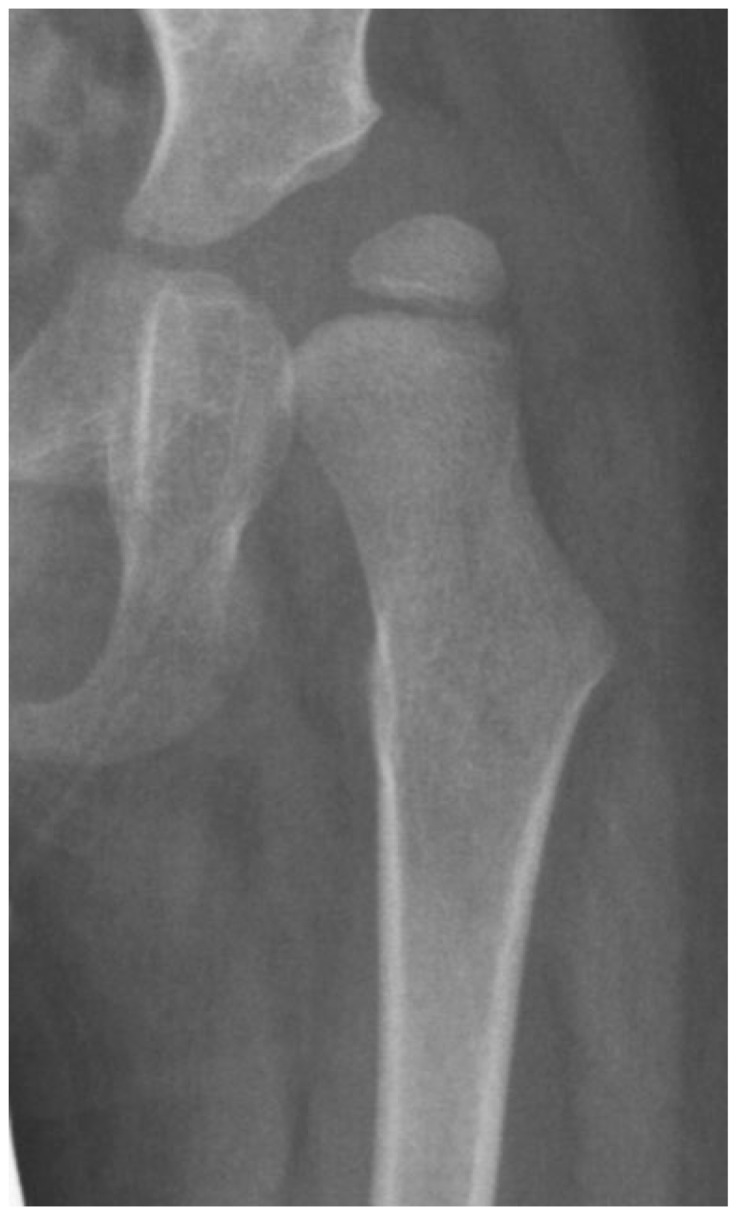
This 3-year-old child with Gross Motor Function Classification System level IV cerebral palsy demonstrates the typical coxa valga, eccentric femoral epiphysis formation, and lateral opening of the acetabulum due to hip joint reaction force focused on the lateral aspect of the acetabulum and the medial aspect of the femoral head.

Our current understanding of how the forces shape the proximal femur are based on the principles first described by Julius Wolff in the 1800s and widely known as Wolff’s law. The principle behind Wolff’s law is that the bone will respond by remodeling and increasing size to sustain the load it experiences [24]. Another observation has been that the epiphyseal growth plate will tend to align to reduce shear stress as long as the force magnitude is within the physiologically sustainable range [25,26]. Bone growth responds to symmetrically distributed compressive stress over the areas of the growth plate. By extension, ossification in the epiphysis occurs at the center of the maximum compressive force in the cartilage [25]. This would explain the eccentric ossification of the capital femoral epiphysis often seen in children with neurologic hip dysplasia (Figure 6).

**Figure 7:**
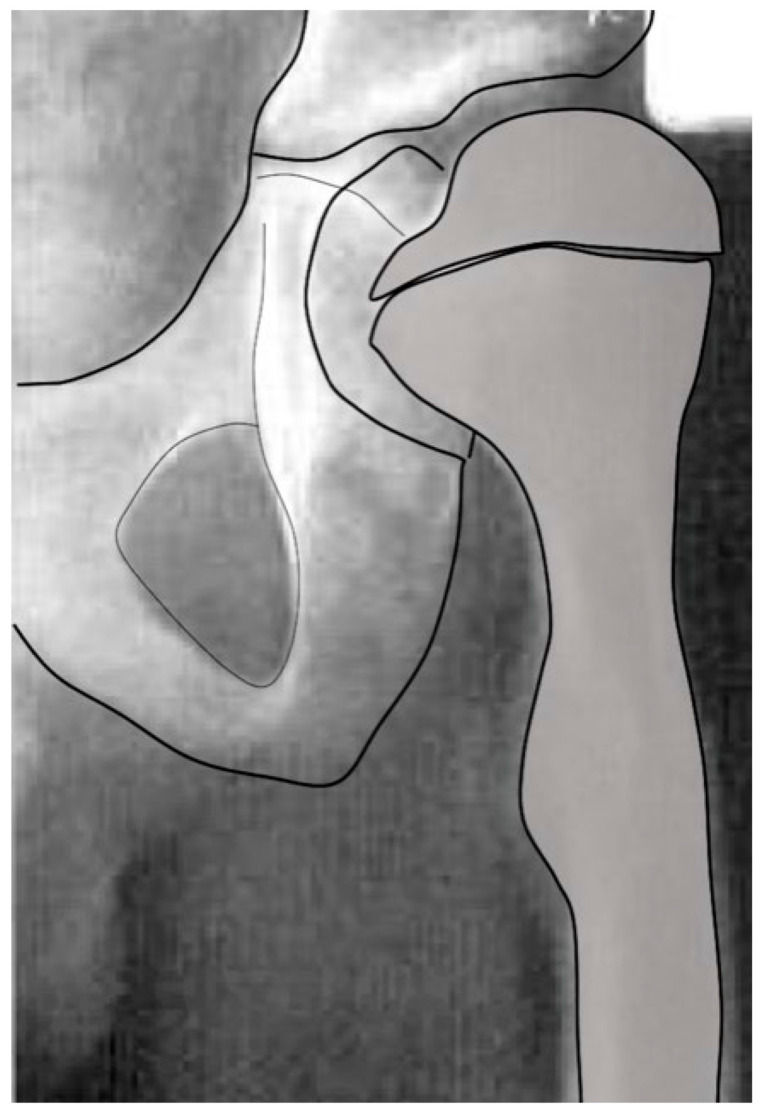
Due to a malignancy in this young male child, at the age of 5 months, the whole abductor medius and minimus was resected along with partial tensor fascia lata and gluteus maximus. At age 15 months, femoral valgus was first seen, and gait was reported to be wide based. This image shows the severe coxa valga 6 years after tumor resection. At this age, this child has no active hip abduction. (Used with permission from Lippincott Williams & Wilkins [23]) “The Creative Commons license does not apply to this content. Use of the material in any format is prohibited without written permission from the publisher, Wolters Kluwer Health, Inc. Please contact permissions@lww.com for further information”.

Another biomechanical principle, the Hueter–Volkmann law, is that the compression side of a bone will reduce growth, and the side under tension or less compression will increase growth. This principle has been extensively tested in animals and is often applied to modeling deformities like idiopathic scoliosis and Blount’s tibia vara [27]. Applying this principle to the proximal femur is difficult because the lateral side of the proximal femur is an apophyseal growth plate, and the medial side is an epiphyseal growth plate with the area on the superior femoral neck being important for proximal femoral longitudinal growth (Figure 1 and Figure 4). Although there have been many modeling studies with finite element analysis, most have focused on the epiphysis and ignored the apophysis [21,25]. Only modeling by Yadev et al. has suggested the important role of trochanteric growth in the proximal femoral shape [22,26]. There are case reports of abductor muscle resections for malignancy in young children that show that the absence of abductor muscles leads to severe coxa valga with reduced lateral femoral growth (Figure 7) [23]. It is not clear if this extreme coxa valga is due to the lack of abductor tension on the apophysis or a response to the joint reaction force from the child’s need to lurch (Supplementary Table S1) over the hip joint in stance phase. Most likely, it is a combined response to both pathologic forces.

**Figure 8:**
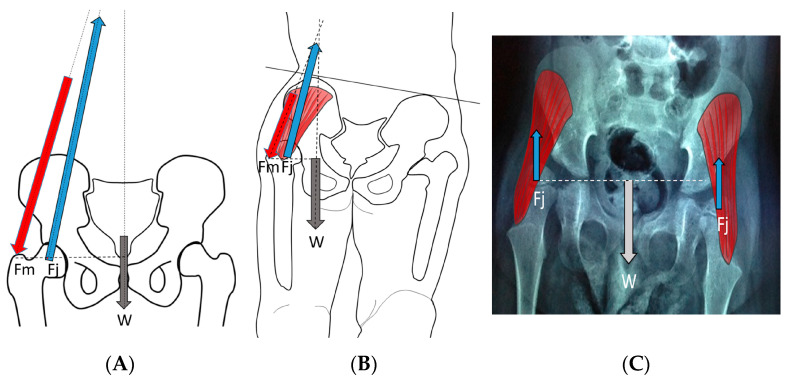
In the normal child (**A**), the primary weight-bearing activity that impacts femoral development occurs during the stance phase of gait and creates a hip joint reaction vector (A [Fj]) directed into the acetabulum. This resultant summated force (A [Fj]) is due to body weight moment (A [W]), which must be balanced by the abductor force (A [Fm]). In the weak or hypotonic ambulatory child whose trunk lurches over the weight-bearing hip during gait (**B**), the abductor force (B [Fm]) is significantly reduced, thereby reducing the hip joint reaction force (B [Fj]), and the hip joint reaction force is also more vertical (B [Fj]). In the child whose summated primary weight-bearing activity is static standing (**C**), the femoral joint reaction force (C [Fj]) is even smaller, approximately half the body weight (C [W]) and completely vertical (C [Fj]).

The hip joint reaction force (Supplementary Table S1) vector is the result of the forces applied to the femoral head through the acetabulum. This force may be modeled as joint forces distributed over the femoral head surface or as a summary vector with an angle of application to the femoral head. This summated vector (HJRF) may also be decomposed into three components either relative to the pelvic or femoral segments. From a conceptual perspective, we feel the summary vector is easiest to understand with the direction being toward the femoral head when we define the effect on the femoral head, and the same vector magnitude can be visualized in the opposite direction to conceptualize the impact on the acetabulum (Figure 8). The HJRF can be modeled as a single force for a specific function or physical activity. For many modeling studies in children with CP, the stance phase of gait is the most commonly utilized [23,25,28]; however, when studies including adults and DDH are considered, there are other activities included such as stair climbing and sit to stand [21]. The force data used in modeling studies are limited to the single events defined by the study due to the high calculation demands of modeling. The force environment that shapes bone development, however, is the impulse (Definition in Supplementary Table S1), which is hard to measure. Based on clinical observations and the use of hypothetical free body analysis of hip mechanics popularized by Bombelli et al. [29], we can make some assumptions as to the reality of the models and the predicted effects. For children who are nonambulatory and have high tone, the HJRF is primarily driven by the co-contraction of different muscle groups, and the vector direction is determined by the position of the thigh segment (femur) relative to the acetabulum based on results of modeling studies [30]. Modeling found little impact of the NSA or FA on hip dysplasia or subluxation risk [30], which has also been confirmed by a clinical study [31].

During the last pregnancy trimester and after birth, normal babies have periods of vigorous kicking movements that have a positive impact on hip development. Restricting this movement by swaddling, especially in maintaining hip extension, has been shown to increase the risk of abnormal hip development [4]. As children start to walk between 12 and 18 months of age, the HJRF becomes mostly influenced by walking activity. The joint reaction force results from the need to balance the lever arms of the body weight and abductor tension to maintain the pelvis horizonal. In a normal situation, the hip abductors are strong stabilizers in the stance phase and produce a vector that is medially oriented (Figure 8). The impact of this medial orientation is to decrease the HSA and NSA of the femur and to deepen the acetabulum. Animal studies in both rabbits [32] and lambs [22] have shown the importance of the direction of the force vector to deepen and enlarge the acetabulum. Modeling has shown that a higher medially directed vector causes increased growth in lateral and central parts of the femoral epiphysis, driving the femoral neck growth into varus and causing the capital femoral growth plate to start to develop a dome shape [33]. Modeling by Heimkes et al. shows the importance of the greater trochanteric apophysis to proximal longitudinal growth of the femur and also contributes to the decrease in the femoral NSA with growth [34,35,36]. More recent finite element modeling simulating walking, jumping, running, and other activities in a growing 6-year-old child found the stress impact in the capital femoral epiphysis was enough to explain the decreasing NSA and FA [22]. The proximal femoral growth plate is continuous with the capital epiphyseal growth plate responding to compressive force and the trochanteric apophyseal plate responding to tension from the gluteal muscles. It is not clear what is the force response of the intermediate growth plate on the superior neck of the femur; however, as the femoral neck increases in valgus with reduced greater trochanter growth, this intermediate growth plate also seems to have reduced growth, and increased longitudinal growth from the capital epiphysis causes an elongated femoral neck that is also thinner (Figure 2).

### 3.3. Abnormal Hip Development in Children with CP and CP-like Conditions

The risk of abnormal hip development leading to hip subluxation and dislocation in children with CP is strongly correlated to the motor ability of the child, as defined by Gross Motor Function Classification System (GMFCS) level [37]. The increased risk of hip subluxation directly correlates to an increase in the GMFCS level of the child, which is an objective measure of gross motor function [5,6,7]. Since there is a large increase in incidence of hip subluxation between GMFCS IV and V compared with GMFCS I–III, we will discuss the differing pathologies of these into two groups, ambulatory or nonambulatory. These separate groups have different outcomes as previously reported [38].

#### 3.3.1. Impact of Abnormal Motor Function on Hip Development in Ambulatory Children

Children with abnormal motor control (Supplementary Table S1), especially those with high tone (Supplementary Table S1) due to spastic CP, will have an increased magnitude and a more vertical hip impulse from muscle co-contraction leading to coxa valga. As the coxa valga increases, the moment arm of the hip abductors decreases, thus requiring increased force to balance the body center of mass. The lurch mechanism brings the body center of mass more directly over the hip joint, changing the force vector to become lower in magnitude but vertical and thus reducing the need for hip abductor force (Supplementary Table S1). The lurching may be so extreme as to require the hip adductors to activate to balance body weight. This lurch leads to increased coxa valga since the capital femoral epiphyseal growth plate tends to align at a right angle following the principle-loading vector to reduce its shear force. The lateral movement of the center of mass makes the hip force vector more vertical (Figure 1), tending to re-enforce and further increasing the NSA and HSA. 

#### 3.3.2. Pathology of Hip Development in Nonambulatory Children

**Figure 9:**
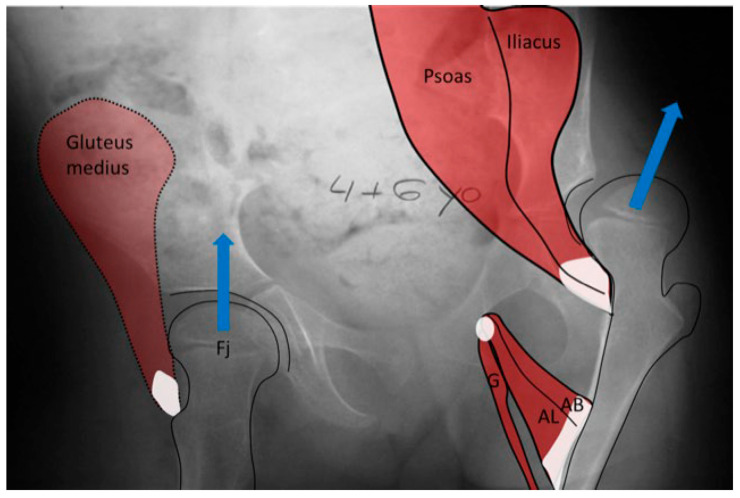
In the child with severe spasticity who is not ambulatory, the summated hip force acting on the proximal femoral shape formation comes from multiple muscles all acting in concert. The hip adductor flexor group (AB, AL, G) tends to have more spasticity, creating an adducted and flexed posture. This posture produces the hip joint reaction force vector in the posterosuperior direction, driving the femoral head out of the acetabulum, as well as acetabular dysplasia and valgus proximal femur due to the growth plate aligning at a right angle to this hip joint reaction force as a way to reduce shear stress. Some hips, however, tend to fall more into abduction, external rotation, and extension at which point the abductor, extensors, and external rotators of the hip develop a mechanical moment arm advantage, thereby propagating this posture. The abducted hip has a high hip joint reaction force vector directed at the central acetabulum aligned along the shaft of the femur, also causing increased coxa valga.

Children with CP GMFCS levels IV and V who are nonambulatory but have high muscle tone tend to show higher muscle force from the hip adductors and flexors compared with the abductors and extensors since the adductor–flexor group tends to be stronger and often more spastic. This muscular imbalance leads to the development of predominant hip posture in adduction and flexion. The adducted and flexed posture provides further advantage due to longer moment arm of the hip flexor and adductor muscle groups while similarly disadvantaging the hip abductor and extensor muscles. This predominant adducted posture causes a high hip joint impulse vector whose direction is toward the posterior and superior aspects of the acetabulum, causing acetabular dysplasia. This leads to the commonly seen dysplasia with the opening of the posterosuperior rim of the acetabulum [39] (Figure 9). The predominating limb position is the most important element in defining the HJRF vector direction and magnitude. Compared with the limb position, femoral NSA and torsion have little impact based on modeling studies with co-contracting muscles in the non-weight bearing environment [30]. A clinical study has reported increasing HSA during childhood growth in this cohort at high risk for hip dysplasia [40]. Some hips develop a predominant posture of abduction, external rotation, and extension that may be a part of the windblown syndrome but may occur unilaterally or bilaterally without the contralateral adducted and flexed hip (Figure 9). The abducted hips have a high femoral head joint reaction force aligned with the shaft of the femur [30], which causes severe coxa valga. However, the vector is medially directed into the acetabulum, creating a deep and stable acetabulum, and even occasionally leading to an acetabular protrusio, as we have seen in severely involved patients (Figure 5). The early formation of the ossification center of the capital femoral epiphysis is usually eccentric in children with CP and tends to be more eccentric in the GMFCS IV and V group due to a more vertical orientation of the HJRF vector (Figure 2).

#### 3.3.3. Pathology of Hip Development in Children with Low Tone and Muscle Weakness 

**Figure 10:**
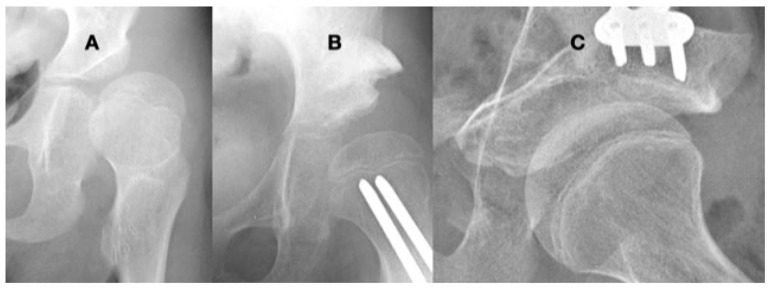
This 9-year-old girl with severe hypotonia, Gross Motor Function Classification System level II, developed anterior hip dislocation. The femoral head is enlarged due to not being contained in the acetabulum but round because she has a full range of motion. The acetabulum is shallow and open globally (**A**). A reconstruction to create anterior lateral coverage with a (Pemberton-type) pelvic procedure was not able to provide sufficient coverage (**B**). By age 13 years, she developed multidirectional instability that was repaired with a periacetabular osteotomy (Bernese-type) (**C**).

**Figure 11:**
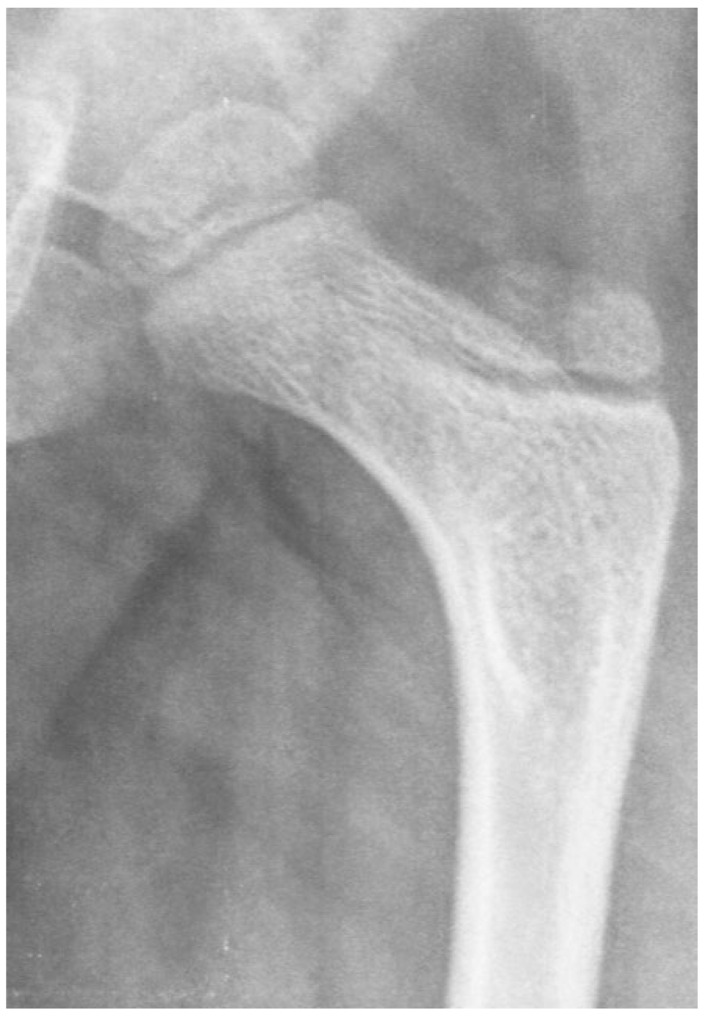
This is an 8-year-old boy with thoracic-level myelomeningocele who is completely flaccid in his lower extremities. He has an infantile-shaped femoral neck shaft angle that has not been affected by weight bearing or muscle force. The acetabulum, however, is very shallow due to the lack of force to stimulate deep socket formation.

There is a group of children with severe hypotonia (Supplementary Table S1) and muscle weakness (Supplementary Table S1) from diverse etiologies who can ambulate. These children tend to have lower magnitude hip joint reaction force vectors, but the hip joint reaction force is often vertically directed due to the lurching gait. The gait patterns of these children have a high degree of trunk shift during weight bearing that keeps the direction of the hip force vector in the acetabulum relatively central if the trunk tilt and pelvic tilt are combined, thereby not causing acetabular dysplasia (Figure 8). However, because the vector magnitude is low, a small shallow acetabulum usually will form (Figure 2). These children are at risk for hip dislocation with the direction being either anterior or posterior since a globally deficient acetabulum is the major component of the hip pathology (Figure 10). Children who are very weak and nonambulatory, such as type I or II spinal muscular atrophy (SMA), also develop severe coxa valga, generally at an earlier age compared with similar high-tone children with CP, but the HSA does not continue to get worse through childhood growth [40]. These babies with SMA are weak but not flaccid at an early age, which likely explains the early development of coxa valga. Patients with flaccid paralysis may have no hip joint force vector and therefore will develop a very shallow acetabulum with poor hip stability, and the HSA may remain at an infantile level of 140–150° since there is no force moderating its growth (Figure 11).

### 3.4. Femoral Factors Influencing Hip Pathology

#### 3.4.1. Pathologic Role of Femoral Anteversion 

The factors that control FA or torsion are not fully understood. From clinical experience, we know that children with motor control problems tend to not resolve the infantile FA [17]. This is presumably due to reduced torsional control driving the correction. Almost every muscle crossing the hip joint creates some torsional moment that varies greatly based on the hip position. These torsional forces tend to be smaller and produced by complex muscle force combinations that make modeling these forces more difficult [25,26,28]. Also, the exact location of this torsion continues to be unclear. In most children, it occurs somewhere between the distal femoral epiphysis and the proximal femur. Thus, the anatomic location of the normal correction of anteversion in growing children has not been well defined. As anteversion increases, finite element modeling has shown an increased force on the more posterior aspect of the femoral head; however, walking with hip flexion causes a higher increase in the posterior hip joint [26,28]. In modeling multiple activities in a 6-year-old child, one study concluded that most activities create forces to decrease FA during growth [22]. Ambulatory children with CP levels GMFCS I–III often have an internal foot and knee progression gait, which causes tripping, awkward gait, and cosmetic concerns. However, FA is not the cause of this gait pattern in all cases [41]. Femoral anteversion is not a predictive factor for hip dysplasia in children with CP [30,31]. Based on population studies, there is less correction of infantile anteversion in nonambulatory children compared with ambulatory children, but there is high individual variation [17,26,42]. In summary, there is much more published research on FA than on coxa valga; however, FA has little direct validated clinical impact on hip joint pathology in children with neurologic disability.

#### 3.4.2. Pathologic Role of Coxa Valga and the Impact of its Correction on Spastic Hips

Based on the current understanding of the mechanics of the hip in children with neurologic motor function problems, hip dysplasia and dislocation tend to be caused by abnormal forces. Normal proximal femoral shape and hip joint development require a good mechanical force environment created through age-appropriate activities of daily living such as walking and running. At skeletal maturity, these abnormal forces have less deforming effect due to inherent bone stability and reduced remodeling at the end of growth. This has been shown in multiple studies of population surveillance of hips in children with CP [5,6,7,43]. These abnormal forces cause coxa valga, acetabular dysplasia, and hip dislocation. Coxa valga has a high correlation to hip dysplasia in children with CP [44], and therefore, the etiology of the hip dislocation may be blamed on the coxa valga [8]. However, these studies usually fail to consider that the hyperabducted side in a windblown deformity has an excessively deep and stable acetabulum but still has severe coxa valga (Figure 5). When a study includes hips with abduction contractures, the correlation of coxa valga to hip displacement decreases [31]. Therefore, treatment should be directed to the primary etiology, which is the abnormal force environment primarily created by the lack of normal physical activity. Although making the physical activity normal is not possible, alterations can be made to the abnormal force environment at the hip. This may also require addressing the secondary pathologies, including coxa valga and acetabular dysplasia.

## 4. Discussion

With an understanding of factors that drive the development of the normal hip and become pathologic factors in neuromuscular hip dysplasia, treatment guidelines can be developed that will improve the outcome. Using selected illustrative cases, the application of these principles will be presented. 

### 4.1. Principles for Management Based on Considering Primary Etiology

#### 4.1.1. Management of the Hip with High Pathologic Force Environment

**Figure 12:**
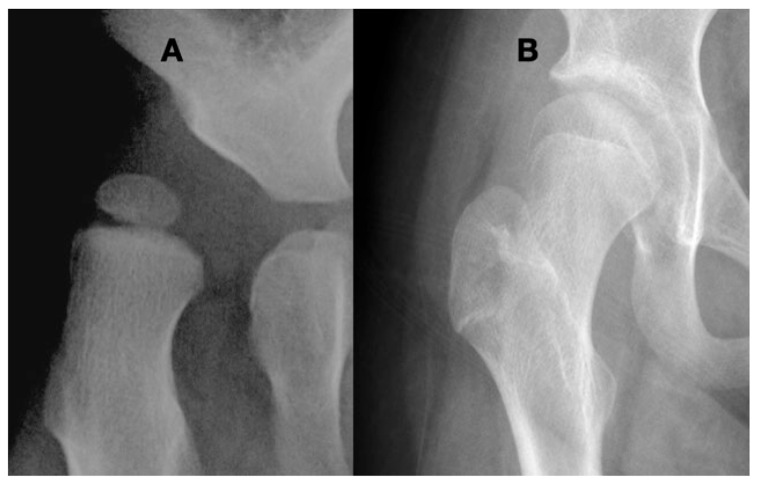
A 2.5-year-old girl was presented at the stage where she was just able to stand in her walker. This is her first screening radiograph (**A**) showing coxa valga, eccentric femoral epiphysis, severe subluxation, and acetabular dysplasia. Infantile hip ultrasounds were reported as normal. The etiology of this hip was felt to be due to her spasticity. Hip adductor lengthening was performed with immediate return to therapy and no splinting or casting. At the age of 14 years, she is at a Gross Motor Function Classification System level III and is walking with crutches, and her hip is well covered but still has a mild superior placement of the acetabulum as evidenced by the break in Shenton’s line (**B**). The only treatment for this hip was force balancing by a muscle-lengthening procedure, which was then enhanced by her increased walking ability as her motor development improved.

For children younger than 6–8 years of age, growth in both the femur and the acetabulum tends to be rapidly evolving. There are also evolving changes in spasticity, reaching a peak at 3–5 years. During this growth period and changing spasticity [43], there is also evolving motor function, which has a longer improvement time in children with GMFCS I and II compared with IV and V [45]. The primary etiology of hip dysplasia in children with high tone is a force that is too high and directed in a posterosuperior direction [30]. At a young age, lengthening or releasing the muscles can balance the force environment, reducing excessive forces and decreasing the predominant position of the leg from adduction, internal rotation, and flexion to a more neutral posture. In children with less severe motor function problems, especially those with the ability to ambulate (GMFCS I–III), the outcome of this procedure at a young age is often successful since motor control also continues to improve [46,47] (Figure 12). For children at GMFCS IV and V, a more extensive muscle release may be required, and the reduced motor control makes predicting the correct amount of release more difficult. In this group of children, there is also less improvement in motor function with increasing age [45]. Although the failure rate is higher, altering the force environment by muscle releases often stabilizes the hip for 2–6 years to an age when there is less risk of recurrent bone deformity following reconstruction [48]. Another alternative is to perform a varus osteotomy, which has the same force ameliorating effect as the adductor lengthening. The varus osteotomy shortens the bone, reducing the force generating ability of the deforming muscle forces. At a young age, recurrent coxa valga is common if there is no concurrent correction of the pathologic force environment. The limited published evidence directly comparing these two approaches suggests that there is no difference in outcome at a follow up after a minimum of 5 years [48].

For children over age 8 years, bone-remodeling potential is progressively decreasing the body’s ability to correct secondary deformities (acetabular dysplasia) following muscle lengthening. However, increasing age also lowers the risk of recurrent bone deformity [49,50]. The correction of coxa valga has positive implications for hips with spastic dysplasia. By performing a varus osteotomy with the removal of a bone wedge, the femur is shortened, and the muscles crossing the hip joint are indirectly lengthened. This lengthening effect on the hip abductors, which are already insufficient compared with the adductors, is not necessarily a positive effect. For this reason, if there is limited hip abduction prior to the osteotomy, a generous release of the hip adductors should be performed to maintain muscle balance. The major benefit of varus osteotomy is providing the hip abductors a better moment arm to be more effective with abduction force generation. The secondary benefit is elevating the greater trochanter with proximal femur varization, which creates a mechanical limitation of the range of hip abduction by impinging on the pelvis, thereby preventing over correction into the hyperabducted hip posture. This effect is equally important for both the adducted and the windblown abducted hips and helps to maintain symmetry. With severe coxa valga, the hip abductor moment arm is very short and becomes completely impotent with moderate adduction. Likewise with coxa valga, moderate hip abduction gives a major advantage to the hip abductors and makes the adductors impotent due to a reduced moment arm. This sensitive imbalance caused by changes in hip position in coxa valga is a major component in the etiology of the windblown hip posture (Figure 10). This muscle force instability due to sensitivity to hip posture is reduced by reducing the coxa valga.

**Figure 13:**
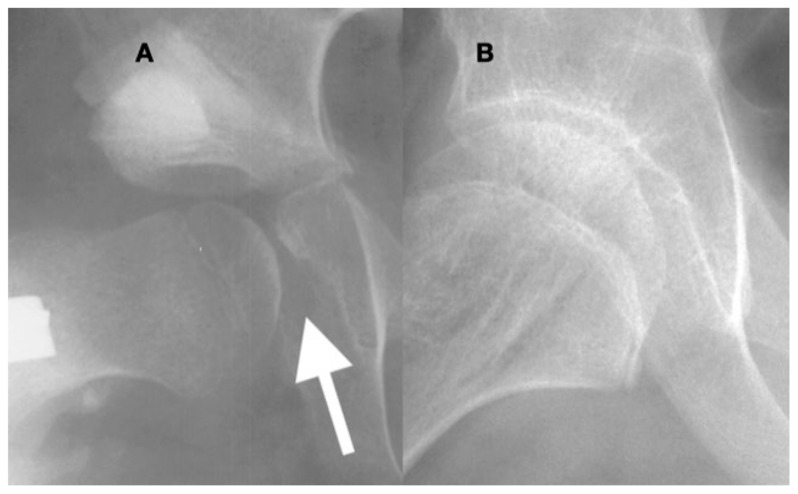
In the acetabulum that has been opened and is then closed with a peri-ilial osteotomy (**A**), the overgrowth of the medial acetabular wall especially in the area of the triradiate growth plate becomes apparent ((**A**), arrow). The acetabular prominence is due to the lack of pressure against the medial wall, and afterward, reduction may prevent the femoral head from seating deep in the acetabulum. In a child such as this 10-year-old, under the influence of femoral head pressure and motion, this prominence can remodel into a congruent acetabulum; however, there is still a thickened medial wall 2 years after reconstruction (**B**).

The amount of coxa valga correction is not well defined in the literature; however, biomechanical studies and moment arm considerations can help to guide us. For children at GMFCS levels I–III with long-term functional ambulation, the goal should be to obtain normal adult values of the NSA with a varus of 120–130°. Avoiding excessive varus will decrease the inactivation of the abductors by their proximal migration. Management goals for patients with a functional gait are normalizing femoral and acetabular anatomy. The ideal age for hip reconstruction is 8–13 years when the bony structure still has the ability to remodel [51] but is not so sensitive to abnormal forces that secondary deformities will recur [49,50]. The femoral head needs to be centered in a stable acetabulum, and to accomplish this, the secondary deformity of the acetabular dysplasia must also be addressed. Failure to address acetabular dysplasia is a risk factor for later failure [52]. The dysplastic acetabulum in the spastic hip is usually of similar size as the femoral head, and it only has an opening acetabular deformity, usually posterosuperior [39]. If the child is younger than 6 years of age, significant acetabular remodeling may occur (Figure 12) [53]; however, with skeletal maturity, the dysplastic acetabulum has less ability to remodel. Based on early developmental studies, hip motion is an important factor for acetabular remodeling [54] in addition to being centered in the acetabulum [16] (Figure 13). This means avoiding significant immobilization that would generate prolonged hip stiffness. For ambulatory patients, it is important to maintain the function of the iliopsoas muscle. If it is released as a part of the varus wedge removal, it should be reattached because an active strong hip flexor is important for going up steps or curbs.

Treatment planning for children with high tone at GMFCS levels IV and V should aim to obtain proper bilateral hip abduction and avoid excessive or asymmetric abduction, which could lead to windblown deformity, increasing the risk of hip subluxation based on modeling studies [30]. Based on these principles, the femoral NSA should be reduced to 90–100°, which is especially important in patients with significant remaining growth [49,50]. This degree of varus can prevent excessive hip abduction and may optimize the moment arm of the abductor muscles. The significant varus also shortens the femur, indirectly lengthening the adductor muscle group. In children with GMFCS levels IV and V, the varus osteotomy should include the removal of the lesser trochanter and insertion of the iliopsoas to release the hip flexors, which act as strong deforming forces and provide no functional benefit in a sitting patient [30].

A component of correcting the NSA valgus is the concomitant correction of the anteversion, which is typically higher than that in age-matched, non-disabled children [17]. Since there is little evidence that femoral anteversion has an implication in either the development of hip dysplasia or coxa valga, the degree of correction should be based on functional goals, with the general aim of obtaining 10–20° of anteversion. Although there is no published literature documenting the importance of avoiding retroversion, it has been the authors’ personal experience that hips set in retroversion have a high risk of recurrent dysplasia. The acetabular dysplasia in hips set in retroversion tends to be directly posterior and is extremely difficult to reconstruct. Soft-tissue releases should be performed if needed to allow anteversion of 0–20° for functional hip rotation.

Children with spasticity who present with painful dislocated hips in late adolescence or adulthood often have severe degenerative changes precluding reconstruction. There are many treatment options with differing risks, complications, and functional outcomes. Because a normal functional hip is not possible from these salvage procedures and we have limited space, we cannot make clear specific recommendations for this group. With improved early screening and treatment, this should become a rare occurrence.

#### 4.1.2. Pathologic Role of Coxa Valga and the Impact of its Correction in Weak and Hypotonic Hips

The primary difference between high-tone (Supplementary Table S1) and weak-hypotonic (Supplementary Table S1) hips is the magnitude of the hip impulse (Supplementary Table S1). Because the HJRF direction is an important aspect in determining the shape of the femur relative to coxa valga [17,26,55], hypotonic hips or hips experiencing low impulse may have even more extreme coxa valga, femoral neck thinning, and elongation compared with the spastic hips. Some of these children have good motor control and can ambulate even with very weak or hypotonic muscles. Therefore, they have significant force exposure that drives valgus formation. The magnitude of the force these hips experience, however, is low and based on Wolff’s law [21], and the bones tend to be small in diameter with large femoral heads and a small shallow acetabulum due to low force directed into the acetabulum [21,32] (Figure 2). Typically, the acetabulum is not misshapen like in the spastic hip but is more globally deficient because the development of depth and size during acetabular formation requires force directed into the acetabulum by the femoral head [21,32]. The femoral head tends to be large due to low force and limited containment in the acetabulum because the femoral head circumferential growth is not restrained by confinement in the acetabulum (Figure 2). However, the femoral head is usually round because these hips often have a very large range of motion, so the hip experiences the whole range of motion, which drives the spherical formation (Figure 2).

Treatment is difficult in hypotonic dysplastic hips with low impulse. There is no role for muscle lengthening to further decrease the force. Reconstruction of the bony secondary changes is the only clinical option and should be considered only if there is a reasonable mechanical force environment such as an ambulating or standing child. Treatment attempts of dislocated hips in severely weak nonambulatory children, such as severe flaccid SMA, are not likely to be successful based on the mechanical understanding that the hip joint reaction force is required to maintain reduction [56]. The hips of less severely involved children with some ambulation tend to have slower progression compared with a higher tone or spastic hips, so careful observation and monitoring in childhood and adolescence are required. The goal is to avoid hips becoming dislocated or severely subluxated. It is best to avoid early reconstruction due to a high rate of recurrent coxa valga and hip instability. Based on biomechanical principles, the reconstruction should bring the NSA angle to 115–125°, and the acetabulum may not be amendable to reshaping procedures because of the large femoral head and shallow short aspect of the ilial portion of the acetabulum. Peri-ilial turn down procedures such as the Dega or Pemberton osteotomies tend to be less effective because they often further reduce the diameter of the acetabulum, which cannot accommodate the enlarged femoral head (Figure 10). Based on considerations of the size discrepancies between the acetabulum and femoral head, acetabular repositioning procedures such as the triple innominate or Bernese osteotomy may be more effective (Figure 10).

#### 4.1.3. Pathologic Role of Coxa Valga and Treatment Options in Paralytic Hips

The pathologic role of coxa valga and the impact of its correction in paralytic hips, which are completely flaccid such as in children with high-level myelomeningocele, have neither internal force from muscular contractions nor external force from weight bearing. Therefore, the hips maintain infantile genetically determined NSA of 130–140° with a very shallow acetabulum (Figure 11). This group includes hips with severe muscle weakness, such as severe type I SMA, but these children often have coxa valga because they had weak movement as infants and in early childhood. Many of these hips remain located although some dislocate due to the absence of force holding the femur to the acetabulum. For children with high-level myelomeningocele or severe SMA, these globally flaccid hips cause few reported problems and need no treatment [38,57]. If degenerative changes develop in myelomeningocele, they are insensate and usually do not become painful. Similar dislocations may occur in children with type I SMA or some other severe muscular dystrophies. Some of these patients develop pain from degenerative changes due to the dislocation. However, with no muscle forces available, success with reconstruction is very unlikely [38,56]. As new treatments such as nusinersen, risdiplam, and onasemnogene abeparvovec are more widely used, these patients with severe SMA may have much better strength [58], which may thereby impact the incidence of hip dislocation, the pathologic presentation, the anatomic pathology of the hip, and the response to treatment. 

The more difficult hips to consider are those with partial paralysis such as mid-level myelomeningocele at L2-4 involvement. These patients may have good hip flexor and quadriceps strength and function; however, they lack hip abductor function, which is key to creating an HJRF vector directed into the acetabulum, providing hip stabilization. Due to the lack of abductor strength, ambulatory patients have a large trunk lurch. Often, the functional muscles are also weak, so the magnitude of the hip total impulse tends to be low with a laterally directed hip joint reaction force vector placing the hip at high risk for recurrent dislocation [30]. However, if these patients are ambulatory, they develop good bone size and coxa valga, although not to the extreme degree of the globally hypotonic patients. Mechanical considerations in treatment planning for these hips are difficult and highly variable. 

### 4.2. New Treatment Options

There has been an increased interest recently in the use of guided growth of the proximal femur as a treatment option for neurologic hip dysplasia [59,60]. This will provide an option for reducing the coxa valga by tethering the inferior aspect of the capital epiphysis or shortening the excessively elongated femoral neck. The impact on hip subluxation is less clear, and the ideal age and severity level are also unclear at this time. 

### 4.3. Limitations

The limitations of this review are due to the published investigations being in many disciplines including embryology, engineering, and orthopedics, often using different terminologies. There is also a limitation in very recent investigations, and some of the publications have only English abstracts, limiting detailed evaluations. 

## 5. Conclusions

The goal of treating the child with neurologic impairment is to prevent hip dysplasia that will affect the child’s function and quality of life. Hip dysplasia develops due to an abnormal force environment during growth. The risk factors for producing this abnormal force environment include motor control, age, muscle tone and force, gross motor function, and predominant thigh posture. These complex factors influence the geometry of the proximal femur with secondary deformities of coxa valga and acetabular dysplasia. These factors increase the risk of hip subluxation and dislocation. 

Normal force environment from normal physical activity during childhood growth is required for normal femoral shape and hip development. These multiple interacting factors when they are abnormal are especially important in early childhood in the development of hip pathology in children with neurologic impairments. 

Children with spasticity develop coxa valga mainly due to a high and vertical hip joint reaction force, which may lead to acetabular dysplasia and hip dislocation. Children with muscle weakness and hypotonia may develop thin femurs with coxa valga, large femoral heads, and a small shallow acetabulum. Treatment planning should consider the individual hip’s mechanical pathology, bone quality, age, and stage of development. 

## Supplementary Materials

The following supporting information can be downloaded at: https://www.mdpi.com/article/10.3390/children11070844/s1. Table S1: Definitions of Terms.

## Abbreviations

HJRF	hip joint reaction force
DDH	developmental hip dysplasia
CP	cerebral palsy
NSA	neck–shaft angle
HSA	head–shaft angle
MP	migration percentage
FA	femoral anteversion
CS	Carnegie stage
GMFCS	Gross Motor Function Classification System
SMA	spinal muscular atrophy

## References

[1] Ito M., Negishi T., Funayama S., Murakami S., Iizuka S. (2024). Early detection and treatment of congenital cataracts using fetal ultrasound: A case of a newborn with a family history of congenital cataracts. Cureus.

[2] Solebo A.L., Rahi J.S., British Congenital Cataract Interest Group (2023). Delayed diagnosis of congenital cataract in preterm infants: Findings from the IoLunder2 cohort study. PLoS ONE.

[3] Wojciech K., Dudek P., Pulik Ł., Łęgosz P. (2024). Screening of developmental dysplasia of the hip in Europe: A systematic review. Children.

[4] Chen X., Liu J., Xue M., Zou C., Lu J., Wang X., Teng Y. (2024). Risk factors of developmental dysplasia of the hip in infants: A meta-analysis based on cohort studies. Orthop. Traumatol. Surg. Res..

[5] Hägglund G., Lauge-Pedersen H., Wagner P. (2007). Characteristics of children with hip displacement in cerebral palsy. BMC Musculoskelet. Disord..

[6] Dobson F., Boyd R.N., Parrott J., Nattrass G.R., Graham H.K. (2002). Hip surveillance in children with cerebral palsy. Impact on the surgical management of spastic hip disease. J. Bone Jt. Surg. Br..

[7] Pruszczynski B., Sees J., Miller F. (2016). Risk factors for hip displacement in children with cerebral palsy: Systematic review. J. Pediatr. Orthop..

[8] Robin J., Graham H.K., Selber P., Dobson F., Smith K., Baker R. (2008). Proximal femoral geometry in cerebral palsy: A population-based cross-sectional study. J. Bone Jt. Surg. Br..

[9] Reimers J. (1980). The stability of the hip in children. A radiological study of the results of muscle surgery in cerebral palsy. Acta Orthop. Scand. Suppl..

[10] Hermanson M., Hägglund G., Riad J., Wagner P. (2015). Head-shaft angle is a risk factor for hip displacement in children with cerebral palsy. Acta Orthop..

[11] Southwick W.O. (1967). Osteotomy through the lesser trochanter for slipped capital femoral epiphysis. J. Bone Jt. Surg. Am..

[12] Van der List J.P., Witbreuk M.M., Buizer A.I., van der Sluijs J.A. (2015). The head-shaft angle of the hip in early childhood: A comparison of reference values for children with cerebral palsy and normally developing hips. Bone Jt. J..

[13] Foroohar A., McCarthy J.J., Yucha D., Clarke S., Brey J. (2009). Head-shaft angle measurement in children with cerebral palsy. J. Pediatr. Orthop..

[14] Ohuma E.O., Papageorghiou A.T., Villar J., Altman D.G. (2013). Estimation of gestational age in early pregnancy from crown-rump length when gestational age range is truncated: The case study of the INTERGROWTH-21st Project. BMC Med. Res. Methodol..

[15] Suzuki Y., Matsubayashi J., Ji X., Yamada S., Yoneyama A., Imai H., Matsuda T., Aoyama T., Takakuwa T. (2019). Morphogenesis of the femur at different stages of normal human development. PLoS ONE.

[16] Watanabe R.S. (1974). Embryology of the human hip. Clin. Orthop. Relat. Res..

[17] Bobroff E.D., Chambers H.G., Sartoris D.J., Wyatt M.P., Sutherland D.H. (1999). Femoral anteversion and neck-shaft angle in children with cerebral palsy. Clin. Orthop. Relat. Res..

[18] Zippel H. (1971). Normal development of the structural elements of the hip joint in adolescence. Beitr. Orthop. Traumatol..

[19] Scorcelletti M., Reeves N.D., Rittweger J., Ireland A. (2020). Femoral anteversion: Significance and measurement. J. Anat..

[20] Pritchett J.W. (1992). Longitudinal growth and growth-plate activity in the lower extremity. Clin. Orthop. Relat. Res..

[21] Vafaeian B., Zonoobi D., Mabee M., Hareendranathan A., El-Rich M., Adeeb S., Jaremko J. (2017). Finite element analysis of mechanical behavior of human dysplastic hip joints: A systematic review. Osteoarthr. Cartil..

[22] Yadav P., Fernandez M.P., Gutierrez-Farewik E.M. (2021). Influence of loading direction due to physical activity on proximal femoral growth tendency. Med. Eng. Phys..

[23] Brien E.W., Lane J.M., Healey J. (1995). Progressive coxa valga after childhood excision of the hip abductor muscles. J. Pediatr. Orthop..

[24] Teichtahl A.J., Wluka A.E., Wijethilake P., Wang Y., Ghasem-Zadeh A., Cicuttini F.M. (2015). Wolff’s law in action: A mechanism for early knee osteoarthritis. Arthritis Res. Ther..

[25] Carriero A., Jonkers I., Shefelbine S.J. (2011). Mechanobiological prediction of proximal femoral deformities in children with cerebral palsy. Comput. Methods Biomech. Biomed. Engin..

[26] Yadav P., Shefelbine S.J., Pontén E., Gutierrez-Farewik E.M. (2017). Influence of muscle groups’ activation on proximal femoral growth tendency. Biomech. Model. Mechanobiol..

[27] Stokes I.A.F. (2002). Mechanical effects on skeletal growth. J. Musculoskelet. Neuronal Interact..

[28] Bosmans L., Wesseling M., Desloovere K., Molenaers G., Scheys L., Jonkers I. (2014). Hip contact force in presence of aberrant bone geometry during normal and pathological gait. J. Orthop. Res..

[29] Bombelli R., Gerundini M., Aronson J. (1984). The biomechanical basis for osteotomy in the treatment of osteoarthritis of the hip: Results in younger patients. Hip.

[30] Miller F., Slomczykowski M., Cope R., Lipton G.E. (1999). Computer modeling of the pathomechanics of spastic hip dislocation in children. J. Pediatr. Orthop..

[31] Chang C.H., Wang Y.C., Ho P.C., Hwang A.W., Kao H.K., Lee W.C., Yang W.E., Kuo K.N. (2015). Determinants of hip displacement in children with cerebral palsy. Clin. Orthop. Relat. Res..

[32] Owiny J.R., Vandewoude S., Painter J.T., Norrdin R.W., Veeramachaneni D.N. (2001). Hip dysplasia in rabbits: Association with nest box flooring. Comp. Med..

[33] Shefelbine S.J., Carter D.R. (2004). Mechanobiological predictions of growth front morphology in developmental hip dysplasia. J. Orthop. Res..

[34] Heimkes B., Posel P., Plitz W., Zimmer M. (1997). Age-related force distribution at the proximal end of the femur in normally growing children. Z. Orthopädie Grenzgeb..

[35] Heimkes B., Posel P., Plitz W. (1995). Biomechanics of the hip joint in children. Z. Orthop. Grenzgeb..

[36] Heimkes B., Posel P., Plitz W., Jansson V. (1993). Forces acting on the juvenile hip joint in the one-legged stance. J. Pediatr. Orthop..

[37] Palisano R.J., Hanna S.E., Rosenbaum P.L., Russell D.J., Walter S.D., Wood E.P., Raina P.S., Galuppi B.E. (2000). Validation of a model of gross motor function for children with cerebral palsy. Phys. Ther..

[38] Gose S., Sakai T., Shibata T., Murase T., Yoshikawa H., Sugamoto K. (2010). Morphometric analysis of the femur in cerebral palsy: 3-dimensional CT study. J. Pediatr. Orthop..

[39] Brunner R., Picard C., Robb J. (1997). Morphology of the acetabulum in hip dislocations caused by cerebral palsy. J. Pediatr. Orthop. B.

[40] Ulusaloglu A.C., Asma A., Rogers K.J., Shrader M.W., Graham H.K., Howard J.J. (2022). The influence of tone on proximal femoral and acetabular geometry in neuromuscular hip displacement: A comparison of cerebral palsy and spinal muscular atrophy. J. Child. Orthop..

[41] Simon A.L., Presedo A., Ilharreborde B., Mallet C., Mazda K., Penneçot G.F. (2014). Can turned inward patella predict an excess of femoral anteversion during gait in spastic diplegic children?. J. Pediatr. Orthop..

[42] Cho Y., Park E.S., Park H.K., Park J.E., Rha D.W. (2018). Determinants of hip and femoral deformities in children with spastic cerebral palsy. Ann. Rehabil. Med..

[43] Lindén O., Hägglund G., Rodby-Bousquet E., Wagner P. (2019). The development of spasticity with age in 4162 children with cerebral palsy: A register-based prospective cohort study. Acta Orthop..

[44] Chougule S., Dabis J., Petrie A., Daly K., Gelfer Y. (2016). Is head-shaft angle a valuable continuous risk factor for hip migration in cerebral palsy?. J. Child. Orthop..

[45] Hanna S.E., Rosenbaum P.L., Bartlett D.J., Palisano R.J., Walter S.D., Avery L., Russell D.J. (2009). Stability and decline in gross motor function among children and youth with cerebral palsy aged 2 to 21 years. Dev. Med. Child. Neurol..

[46] Shore B.J., Yu X., Desai S., Selber P., Wolfe R., Graham H.K. (2012). Adductor surgery to prevent hip displacement in children with cerebral palsy: The predictive role of the Gross Motor Function Classification System. J. Bone Jt. Surg. Am..

[47] Presedo A., Oh C.W., Dabney K.W., Miller F. (2005). Soft-tissue releases to treat spastic hip subluxation in children with cerebral palsy. J. Bone Jt. Surg. Am..

[48] Kiapekos N., Broström E., Hägglund G., Åstrand P. (2019). Primary surgery to prevent hip dislocation in children with cerebral palsy in Sweden: A minimum 5-year follow-up by the national surveillance program (CPUP). Acta Orthop..

[49] Davids J.R., Gibson T.W., Pugh L.I., Hardin J.W. (2013). Proximal femoral geometry before and after varus rotational osteotomy in children with cerebral palsy and neuromuscular hip dysplasia. J. Pediatr. Orthop..

[50] Minaie A., Gordon J.E., Schoenecker P., Hosseinzadeh P. (2022). Failure of hip reconstruction in children with cerebral palsy: What are the risk factors?. J. Pediatr. Orthop..

[51] Braatz F., Staude D., Klotz M.C., Wolf S.I., Dreher T., Lakemeier S. (2016). Hip-joint congruity after Dega osteotomy in patients with cerebral palsy: Long-term results. Int. Orthop..

[52] Chen B.J.P., Çobanoğlu M., Sees J.P., Rogers K.J., Miller F. (2023). Recurrent hip instability after hip reconstruction in cerebral palsy children with spastic hip disease. J. Orthop. Sci..

[53] Chang F.M., Ma J., Pan Z., Ingram J.D., Novais E.N. (2016). Acetabular remodeling after a varus derotational osteotomy in children with cerebral palsy. J. Pediatr. Orthop..

[54] Nowlan N.C., Chandaria V., Sharpe J. (2014). Immobilized chicks as a model system for early-onset developmental dysplasia of the hip. J. Orthop. Res..

[55] Yadav P., Shefelbine S.J., Gutierrez-Farewik E.M. (2016). Effect of growth plate geometry and growth direction on prediction of proximal femoral morphology. J. Biomech..

[56] Sporer S.M., Smith B.G. (2003). Hip dislocation in patients with spinal muscular atrophy. J. Pediatr. Orthop..

[57] Thompson R.M., Foley J., Dias L., Swaroop V.T. (2019). Hip status and long-term functional outcomes in spina bifida. J. Pediatr. Orthop..

[58] Carson V.J., Young M., Brigatti K.W., Robinson D.L., Reed R.M., Sohn J., Petrillo M., Farwell W., Miller F., Strauss K.A. (2022). Nusinersen by subcutaneous intrathecal catheter for symptomatic spinal muscular atrophy patients with complex spine anatomy. Muscle Nerve.

[59] Hsu C.M., Sheu H., Lee W.C., Kao H.K., Yang W.E., Chang C.H. (2023). Soft tissue releases with simultaneous guided growth decrease risk of spastic hip displacement recurrence. J. Pediatr. Orthop..

[60] Lebe M., van Stralen R.A., Buddhdev P. (2022). Guided growth of the proximal femur for the management of the ‘hip at risk’ in children with cerebral palsy-a systematic review. Children.

