# Supplementary material for: The Etiology of Neuromuscular Hip Dysplasia and Implications for Management: A Narrative Review
Source: Children (Basel). 2024 Jul 11;11(7):844. doi: 10.3390/children11070844 (PMC11275045; doi:10.3390/children11070844)
Supplement: Supplementary file 1 [file children-11-00844-s001.zip › children-3079559-supplementary.pdf]

Supplemental Table S1. Definitions of Terms

Term	Definition
Femoral anteversion	The mechanical definition is internal rotation of the distal femur defined by the axis of the knee joint relative to the proximal femur. A common anatomical definition is the anterior projection of the femoral neck relative to the transverse plane of the distal femur. Synonyms include internal femoral torsion and inclination of the femoral neck.
Femoral retroversion	The opposite of anteversion, defined as external rotation of the distal femur relative the proximal femur. Synonyms include external femoral torsion and femoral neck declination.
Reimers' migration percentage	The percentage of the femoral head that is lateral to the lateral edge of the acetabulum. This is the primary measurement of hip subluxation in children with neurologic impairments.
Femoral head-shaft angle	The radiological angle between the shaft of the proximal femur and a line perpendicular to the proximal femoral epiphysis on an anteroposterior radiograph of the pelvis.
Acetabular size	Since the normal acetabular shape is a partial sphere, size may be measured as the volume. A more common measurement is the acetabular depth measured as the distance from a line between two edges of the acetabulum to the medial wall. The edge of the acetabulum is irregular when assessed by computed tomography scan; therefore, objective measures of depth depend on the imaging used and the technique for defining the edge points.
Dysplasia	Hip dysplasia is defined as abnormal growth and development of the hip with no reference as to the specific deviation.
Impulse	This mechanical term means the summation of a force applied over a duration of time (integral of force over time). This means the impulse is a vector with direction and magnitude.
Motor control	The ability to voluntarily contract a single muscle without initiating contraction in other muscles is normal motor control. Loss of motor control often leads to co-contraction of opposing muscle groups or developing aberrant joint postures or movements.
Hip joint reaction force	This is the common term used to define the resultant force environment of the hip joint. The classic free body analysis of the hip focused on the period of single leg stance of gait. This force is a combination of the muscle forces required to balance the body over the hip and the ground reaction force balancing the body mass. This is vector force with direction and magnitude, and can be decomposed into vertical, medial-lateral, and anteroposterior components.
Hip joint impulse	The hip may experience many different types of force throughout a day. In normal patients, the largest component is likely the combined forces experienced during single-leg stance. However, in children with an abnormal gait or who are nonambulatory, the hip force summated over time may be mostly generated by muscle co-contraction or low-level muscle force and atypical posture such as hip

	adduction and flexed posture. In this way, a lower force applied over a much longer time can generate a higher hip impulse. Based on current bone response theory, the hip impulse is the factor that drives hip dysplasia in neuromuscular impairments.
Muscle weakness	Muscle strength is defined relative to the maximum force generation a single muscle can generate. Therefore, muscle weakness means the muscle is not able to generate the maximum force expected based on the age and size of the child.
Hypertonic muscles, spasticity, dystonia	In this paper, where our main goal is to define the impulse generation of the hip joint reaction force, we consider these terms to be similar. These terms are not synonymous as there are clearly patients who have more or less of these movement disorders, but most children with upper motor neuron lesions have a mixed movement disorder. From the perspective of generating hip impulse, all these movement disorders have some level force generation, usually considerably less than maximum magnitude for age-matched children but applied over a much longer time frame each day. The other aspect of the hypertonic state is usually muscle length decrease over time limiting the range of motion over which the hip impulse functions, leading to what is commonly termed muscle contractures.
Hypotonia	This is the opposite of hypertonia and muscles are usually weak; however, there tends to be an exaggerated range of motion and the muscles have less ability to generate maximum force and are active for shorter time periods. Therefore, the impulse magnitude is greatly reduced compared with the hypertonic state, but the vector direction is more variable due to large range of motion.
Lurch	As a technical gait term it means moving the upper body (center of mass) laterally over the weight-bearing limb. This movement greatly reduces the hip joint reaction force by reducing the abductor muscle force required, thereby also reducing the pressure in the hip joint. It is a common maneuver used to reduce pain in an arthritic hip or to compensate for abductor weakness. Although the magnitude of the hip joint reaction force is reduced, the vector direction is much more vertical compared with single-leg stance in normal gait. A synonymous term is compensated Trendelenburg gait.
Trendelenburg	Many tests and devices are named after this famous German surgeon, Friedrich Trendelenburg (1844-1924), who had many prominent students who named things after him, therefore creating confusion as to the meaning of specific physical examination findings. Some examples of these include the following (based on Wikipedia, current November 2021).<br>Trendelenburg sign – In static stance, the pelvis drops on the non-weight-bearing side when the patient is asked to stand on one leg, indicating abductor weakness.<br>Trendelenburg gait – The same posture as the sign occurs with drop of the swing side of the pelvis.

	Compensated Trendelenburg gait – The upper body shifts over the weight-bearing limb to compensate for abductor weakness, synonymous with lurching for weakness.<br>Trendelenburg position – Patient in supine position with the table reclined cranial (head) end down 30°.<br>Trendelenburg test – The leg is raised above the level of the heart with the patient supine to test that varicose veins empty as a check of vein valve function.
